# Effect of rosuvastatin on outcomes in chronic haemodialysis patients – design and rationale of the AURORA study

**DOI:** 10.1186/1468-6708-6-9

**Published:** 2005-05-23

**Authors:** Bengt Fellström, Faiez Zannad, Roland Schmieder, Hallvard Holdaas, Alan Jardine, Helen Rose, Wim Wilpshaar, the AURORA Study Group

**Affiliations:** 1 Department of Medical Science, Renal Unit, University Hospital, Uppsala, Sweden; 2 Clinical Investigation Center INSERM (CIC), Hôpital Jeanne d'Arc, Toul, France; 3 Med Klinik IV, Univ.-Klinik Erlangen-Nürnberg, Germany; 4 Department of Nephrology, Rikshospitalet, Oslo, Norway; 5 Department of Medicine and Therapeutics, Western Infirmary Hospital, Glasgow, United Kingdom; 6 AstraZeneca, Macclesfield, United Kingdom; 7 AstraZeneca, Macclesfield, United Kingdom

**Keywords:** atherosclerosis, cardiovascular disease, end-stage renal disease, haemodialysis, lipids, statin

## Abstract

**Background:**

Patients with end-stage renal disease (ESRD) are at high risk of cardiovascular events. Multiple risk factors for atherosclerosis are present in ESRD and may contribute to the increased risk of cardiovascular mortality in this population. In contrast to patients with normal renal function, the benefits of modifying lipid levels on cardiovascular outcomes in patients with ESRD on haemodialysis have yet to be confirmed in large prospective randomised trials. A study to evaluate the Use of Rosuvastatin in subjects On Regular haemodialysis: an Assessment of survival and cardiovascular events (AURORA) will be the first large-scale international trial to assess the effects of statin therapy on cardiovascular morbidity and mortality in ESRD patients on chronic haemodialysis.

**Methods:**

More than 2,750 ESRD patients who have been receiving chronic haemodialysis treatment for at least 3 months have been randomised (1:1), irrespective of baseline lipid levels, to treatment with rosuvastatin 10 mg or placebo. The primary study endpoint is the time to a major cardiovascular event (first occurrence of cardiovascular death, non-fatal myocardial infarction or non-fatal stroke). Secondary endpoints include all-cause mortality, major cardiovascular event-free survival time, time to cardiovascular death, time to non-cardiovascular death, cardiovascular interventions, tolerability of treatment and health economic costs per life-year saved. Study medication will be given until 620 subjects have experienced a major cardiovascular event.

**Conclusion:**

Our hypothesis is that results from AURORA will establish the clinical efficacy and tolerability of rosuvastatin in patients with ESRD receiving chronic haemodialysis and guide the optimal management of this expanding population.

## Background

Patients with end-stage renal disease (ESRD) undergoing haemodialysis have substantially higher cardiovascular disease mortality rates than the general population [1-5]. Accelerated atherosclerosis has been observed in haemodialysis patients [6] and may contribute to this increased cardiovascular event rate. Treatment of ESRD and its cardiovascular consequences places a large burden upon healthcare providers, and the cost and prevalence are expected to increase greatly over the next decade [5]. Therefore, controlling risk factors for atherosclerosis may play an important role in preventing cardiovascular events in these individuals.

Hydroxy-methylglutaryl-coenzyme A (HMG-CoA) reductase inhibitors (statins) have been demonstrated to reduce coronary heart disease morbidity and mortality in several landmark trials [7-14]. The reductions in cardiovascular events also occur in patients with average to lower than average baseline low-density lipoprotein cholesterol (LDL-C) levels [12,14] and the benefits of statin therapy can be independent of lipid lowering [15]. Most haemodialysis patients do not have elevated cholesterol (total and LDL-C) [16,17], and the highest mortality risk is often in patients with very low cholesterol levels [18]. Other chronic diseases and malnutrition may act to lower blood cholesterol and independently increase the risk of death, thereby contributing to the negative association between very low cholesterol and mortality observed in these patients [19,20]. Indeed, a recent prospective study suggests that levels of total cholesterol (TC) may be associated with mortality in haemodialysis patients without evidence of inflammation and malnutrition [20]. Haemodialysis patients have other lipid abnormalities such as lower levels of high-density lipoprotein cholesterol (HDL-C) and elevated intermediate-density lipoprotein (IDL) and triglycerides [16,17].

Although the efficacy of statins is well established in conditions associated with increased cardiovascular risk, dialysis patients have generally been excluded from statin outcome trials because of their related co-morbidities and as a result of pharmacokinetic and safety issues. It is not appropriate to extrapolate evidence from patients with normal renal function to patients with ESRD on haemodialysis, and there is a need to investigate the benefit-risk profile of statins specifically in this population. Data from an observational study indicate that statin treatment may improve survival in patients with ESRD [21]. Mortality was 32% lower in patients with ESRD who received statin treatment compared with patients not receiving statins, a finding that is consistent with other large outcome statin trials [7,8,11]. However, observational studies do not establish a causal relationship and large-scale randomised studies are required [22]. The Die Deutsche Diabetes Dialyse Studie (4D study) investigated the effect of atorvastatin 20 mg or placebo on cardiovascular mortality, non-fatal myocardial infarction and stroke in 1,255 patients with type 2 diabetes who were on haemodialysis treatment for no more than 2 years [23]. Initial results presented at the annual meeting of the American Society of Nephrology indicate that the primary endpoint was reduced by 8% after 4 years' treatment with atorvastatin, but this was not significantly different from placebo [24]. Further large-scale long-term prospective, randomised studies are required to investigate the efficacy and safety of statins for reducing cardiovascular morbidity and mortality, specifically in ESRD patients on haemodialysis [22].

Rosuvastatin is the most efficacious of the available statins for lowering LDL-C levels, causing reductions of 47% at the initial starting dose of 10 mg [25]. In addition, rosuvastatin has benefits across the lipid profile, including increases in HDL-C, reductions in small dense LDL and triglyceride-rich lipoprotein particles [25,26], and has a safety profile consistent with other available statins [27]. These properties make it an ideal agent for a study investigating the benefits of statin treatment for the prevention of cardiovascular events in a population of patients at high risk of cardiovascular events. AURORA (A study to evaluate the Use of Rosuvastatin in subjects On Regular haemodialysis: an Assessment of survival and cardiovascular events) is the first large-scale international trial to assess the effects of a statin on cardiovascular morbidity and mortality in ESRD patients on chronic haemodialysis irrespective of baseline lipid levels.

## Methods

### Study design

AURORA is a double-blind, randomised, multicentre, phase IIIb, parallel-group study comparing the effects of rosuvastatin (10 mg once daily) with placebo on survival and cardiovascular events in ESRD patients on chronic haemodialysis.

**Figure 1:**
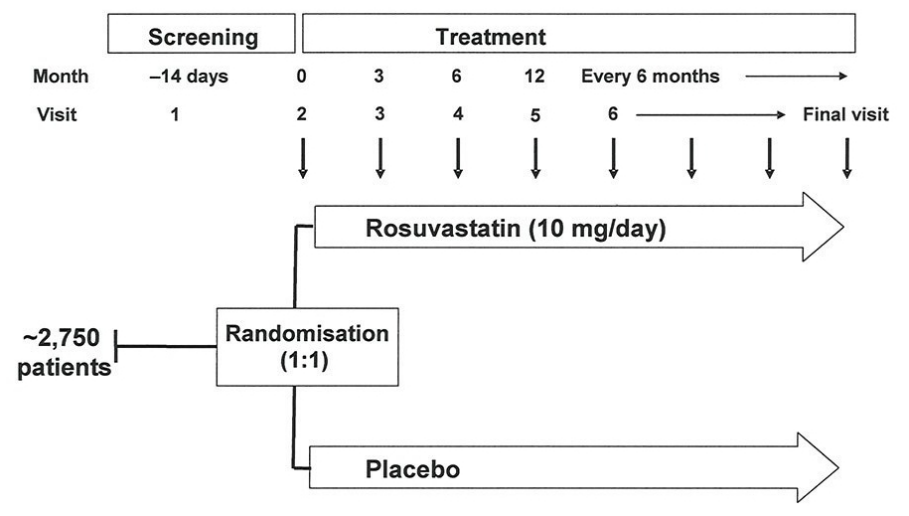
Study design

More than 2,750 subjects from approximately 300 centres in Europe, Canada, Australia, Brazil, Mexico and South Korea have been randomised, irrespective of baseline lipid levels. During the recruitment period, patients were screened over 2 weeks for eligibility according to the major inclusion and exclusion criteria (Table 1). Demographic data were collected on age, gender, race, medical history, physical examination (including height, weight, blood pressure and heart rate), smoking status, and method and duration of dialysis (Table 2). Patients were then randomised (1:1) into the two blinded treatment arms of the study, rosuvastatin 10 mg/day or placebo, and visits to assess safety and efficacy are scheduled to occur at 3 months, 6 months and every 6 months thereafter until the end of the study (Figure 1; Table 2).

**Table 1:** Major eligibility criteria

**Inclusion**
Men and women aged 50–80 years
End-stage renal failure and chronic haemodialysis for at least 3 months
Provision of written informed consent
**Exclusion**
Underlying haematological, neoplastic, GI, metabolic (other than diabetes) or infectious condition expected to reduce survival to less than 1 year
Patients likely to require a kidney transplant within 1 year
Statin therapy within the previous 6 months
History of serious reactions to statins
Unexplained CK >3 times ULN
Active liver disease (ALT >3 times ULN)
Uncontrolled hypothyroidism
A disallowed medication, such as another lipid-modifying agent or cyclosporin

**ALT: **Alanine transaminase, **CK: **Creatine kinase, **GI: **Gastrointestinal, **ULN: **Upper limit of normal

#### Objectives and endpoints

The primary objective of the AURORA study is to compare the effects of rosuvastatin with placebo on cardiovascular events. The primary endpoint is the time to a major cardiovascular event (cardiovascular death, fatal myocardial infarction or non-fatal stroke). An independent and blinded Clinical Endpoint Committee (see Appendix) will review all deaths, strokes and myocardial infarctions using a predefined set of event definitions to ensure consistency of event diagnosis across all subjects throughout the study.

Secondary endpoints include all-cause mortality, cardiovascular event-free survival, cardiovascular death, non-cardiovascular death, procedures as a result of stenosis or thrombosis of the vascular access for chronic haemodialysis (arteriovenous fistulas and grafts only), and coronary or peripheral revascularisations. Furthermore, the tolerability of rosuvastatin in ESRD patients undergoing regular chronic haemodialysis will be compared with placebo. The health economic impact of rosuvastatin treatment on the utilisation of resources and costs associated with the occurrence of a major cardiovascular event will also be assessed. Costs related to hospitalisations will be combined with survival analyses to enable the calculation of cost per life-year saved.

Tertiary objectives include assessing the efficacy of treatment at 3 and 12 months post-randomisation on high-sensitivity C-reactive protein (hsCRP) and various fasting lipid parameters: TC, LDL-C, HDL-C, non-HDL-C, TC/HDL-C, LDL-C/HDL-C, triglycerides, apolipoprotein B (Apo B), apolipoprotein AI (Apo AI), Apo B/Apo AI ratio, and oxidised LDL. Additionally, changes in TC, LDL-C, HDL-C, non-HDL-C, TC/HDL-C, LDL-C/HDL-C and triglycerides will be assessed after 24 months, 36 months (then yearly as required) and at the final visit. A central laboratory service (Quintiles Laboratories, Livingston, UK) certified for standardisation of lipid analyses by the Standardization Program of the Center for Disease Control and Prevention and the National Heart, Lung and Blood Institute will perform all laboratory testing of lipids and hsCRP. Additional laboratory work will be performed in subgroup studies analysing the predictive power of various other markers of cardiovascular risk.

Genetic research may also be performed on certain subjects at some study sites on an optional basis. DNA samples will be obtained for future research into the effects of genetic polymorphisms on the response to rosuvastatin and placebo. Susceptibility to, and prognosis of, cardiovascular disease and lipid disorders will also be studied.

### Study population

#### Eligibility and treatment withdrawal

Irrespective of baseline lipid levels, patients aged 50–80 years with ESRD who have been receiving chronic haemodialysis for at least 3 months were considered for inclusion in the study unless they fulfilled any of the exclusion criteria (key criteria detailed in Table 1). In addition, patients who undergo kidney transplant surgery will be withdrawn from study medication, but will continue to follow scheduled study assessments. Treatment will also be discontinued if patients have creatine kinase >10 times upper limit of normal (ULN) accompanied by unexplained muscle pain or if myopathy is suspected or if the patient becomes pregnant. Other reasons that may lead to treatment discontinuation include: an adverse event or endpoint that in the opinion of the investigator warrants treatment withdrawal; alanine transaminase increase >3 times ULN on two occasions; other safety reasons as judged by the investigator; protocol non-compliance.

#### Ethics

The study protocol and patient consent form have been approved by an Independent Ethics Committee or Institutional Review Board, as appropriate. The study is being performed in accordance with the ethical principles that have their origin in the Declaration of Helsinki and are consistent with International Conference on Harmonisation/Good Clinical Practice. The investigator from each centre will ensure that subjects are given full and adequate oral and written information about the nature, purpose, risks and benefits of the study and that written consent is obtained. The conduct and progress of the study is reviewed by the Steering Committee and Executive Steering Committee (see Appendix). In addition, an independent Data and Safety Monitoring Board (DSMB) (see Appendix) is responsible for monitoring all safety aspects of the study. The DSMB will review and evaluate all serious adverse events and endpoints at least every 6 months using unblinded data and will then report to the Executive Steering Committee in a blinded manner. The DSMB will also be responsible for making recommendations to the Executive Steering Committee regarding modifying or stopping the study early.

### Data analysis

The efficacy endpoints will be analysed using the intent-to-treat population, including all randomised subjects. For the time-to-event endpoints, Kaplan-Meier curves will be used to show the proportion of subjects without an endpoint. An unadjusted Cox Proportional Hazards model with treatment as the covariate will be used to analyse the endpoint. An additional Cox Proportional Hazards regression model [28] will also be constructed adjusting for known and potential risk factors as a secondary analysis and to test for interactions between risk factors and treatment. The number of coronary or peripheral revascularisations will also be summarised, but not formally analysed. For the tertiary endpoints, the percentage change from baseline for each parameter will be assessed for each time point by an analysis of covariance, with the baseline value as a covariate. An exploratory analysis will also be conducted to examine whether lipid values at 3, 12, 24, 36 months (yearly if required) and at the final visit are predictive of future cardiovascular events.

The sample size was determined such that the study will be adequately powered for the analysis of the primary endpoint, time from randomisation to a major cardiovascular event. Based on previous studies, it is anticipated that the cardiovascular event rate in the placebo group will be 11% per year. The sample size has been calculated in order to be able to detect a 25% reduction in cardiovascular events rate per year at a two-sided significance level of 4.86%, with 90% power. The study will continue until 620 subjects have experienced a major cardiovascular event. This is expected to be approximately 3.9 years after initiation of the study, which began in January 2003. An interim efficacy analysis will be conducted by the DSMB when 310 subjects have experienced a major cardiovascular event. The purpose of the interim analysis is to investigate, at this time, the possibility of concluding a statistically significant difference between the treatments for time from randomisation to major cardiovascular event.

## Discussion

Patients with ESRD have an increased risk of cardiovascular morbidity and mortality [2,4], and this contributes to the large burden this disease places upon healthcare providers [5]. A number of studies have indicated that patients on dialysis with very low TC levels have a higher rate of mortality [18,20]. Both small dense LDL and triglyceride-rich lipoproteins have been implicated in the development of cardiovascular disease [29-32] and are common in patients with ESRD and on haemodialysis [16,33-37]. Triglyceride-rich lipoproteins, in particular IDL, are prevalent and predictive of atherosclerosis in haemodialysis patients [17]. Thus, modifying lipid levels with a statin should have beneficial effects on cardiovascular outcomes in these patients. Large outcome studies also indicate that the positive effects of statins occur irrespective of baseline lipid levels [12,14]. While the efficacy of statins for altering the lipid profile has been studied previously in ESRD patients [38-42], their long-term benefits on cardiovascular outcomes in this population have not yet been examined and dialysis patients have generally been excluded from statin outcome trials.

Several studies examining outcome after statin treatment have recently been completed or are in progress in patients with related conditions. In a study in renal transplant patients, fluvastatin reduced the risk of cardiac death or non-fatal myocardial infarction by 35% compared with subjects receiving placebo (risk ratio 0.65 [95% confidence intervals 0.48–0.88]; p = 0.005) [43]. Initial findings of the 4D study did not demonstrate a significant difference between atorvastatin 20 mg and placebo treatment; however, LDL-C levels were reduced during the study period [24]. Another study is ongoing; the Study of Heart And Renal Protection (SHARP) aims to assess the effects of simvastatin and the cholesterol-absorption inhibitor ezetimibe among patients with chronic kidney disease [18]. AURORA is the first international, prospective, randomised, double-blind, placebo-controlled study to assess whether statin therapy alone can reduce the incidence of coronary events in ESRD patients on chronic haemodialysis.

Most haemodialysis patients do not have elevated LDL-C and, in accordance with findings from the Heart Protection Study, the AURORA trial will randomise patients irrespective of their baseline lipid levels [12]. Patients on haemodialysis have lower levels of HDL-C and higher triglycerides compared with control subjects [16]. Accordingly, a statin with efficacy across the lipid profile would be appropriate to assess the benefits of treatment in ESRD patients. Rosuvastatin is the most efficacious statin reported for lowering LDL-C [44], and has benefits across the lipid profile. In a study of hypercholesterolaemic patients, rosuvastatin has been shown to increase HDL-C by 7.7–9.6% compared with 4.4–5.7% for atorvastatin and 5.2–6.0% for simvastatin across the 10–40 mg dose range [44]. In addition, rosuvastatin has been shown to substantially lower triglyceride-rich lipoproteins in both hypercholesterolaemic and hypertriglyceridaemic patients [45]. The effects of haemodialysis upon excretion of statins should also be considered. On the basis of pharmacokinetic, pharmacodynamic and safety data obtained from a pilot study, rosuvastatin 10 mg can be safely administered to patients with ESRD on chronic haemodialysis (data on file, AstraZeneca).

In addition to their ability to modify lipid levels, statins produce other effects that may benefit ESRD patients. Oxidative stress has been implicated in the pathogenesis of cardiovascular disease, and it is enhanced in patients with renal insufficiency [46,47]. Recently, statins have been reported to have anti-oxidant effects [48], which may be of benefit to these patients. Statins may also have additional non-lipid or pleiotropic effects, for example, improving endothelial function [15]. Endothelial dysfunction has been implicated in the development of cardiovascular disease and has been shown to be closely associated with the degree of renal insufficiency [49]. Pre-clinical experiments have shown that rosuvastatin can improve endothelial function [50], although this has yet to be verified in a clinical situation. Finally, left ventricular hypertrophy and heart failure can also develop in ESRD patients on haemodialysis [51]. Statin therapy has been reported to reduce left ventricular hypertrophy [52] and retrospective analyses indicate that these agents can also help to prevent heart failure [53]. These properties could provide an additional beneficial effect in ESRD patients.

The AURORA study should provide valuable information on the utility of statin treatment for the reduction of cardiovascular events in patients with ESRD. As an atherogenic lipid profile and endothelial dysfunction are often present in individuals with ESRD and contribute to the development of atherosclerosis, it is anticipated that rosuvastatin will improve cardiovascular outcomes in this patient population. Furthermore, it is hoped that AURORA will produce data on the cost effectiveness (cost due to hospitalisation and cost per year of life saved) and long-term safety of rosuvastatin treatment in ESRD patients receiving chronic haemodialysis. This study will also generate a large database of information from a well-documented cohort, which may be valuable in the epidemiological evaluation of cardiovascular risk in patients with ESRD.

## Competing interests

This study is sponsored by AstraZeneca. The authors also have affiliations with other pharmaceutical companies including Pfizer (AJ, BF, HH, RS, FZ), Bristol-Myers Squibb (AJ, HH, RS), Novartis (AJ, BF, HH, RS, FZ), Fujisawa (AJ, BF), Roche (AJ, BF, HH, RS), Merck (AJ, BF, HH, RS), Schering-Plough (BF, HH), Wyeth (AJ, BF), GlaxoSmithKline (AJ, BF, HH), Servier (AJ), Actelion (AJ), Pharmalink (BF). These relate to personal or institutional-affiliated receipt of income in the areas: Research grants, honoraria and Consultant fees presently or during the last five years. The authors WW and HR are employed by AstraZeneca.

## Authors' contributions

Author BF is the Principal Investigator of the study and contributed to the concept and design of the study

Authors FZ, RS, HH, AJ and WW contributed to the design of the study

Author HR contributed to the planned statistical analyses of the study and sample size determination.

All authors read and approved the final manuscript.

## Appendix

### Executive Steering Committee

Professor B Fellström (Principal Investigator; Uppsala, Sweden), Professor F Zannad (Toul, France), Professor R Schmieder (Erlangen, Germany), Dr H Holdaas (Olso, Norway), Dr A Jardine (Glasgow, UK).

### Steering Committee

Executive Steering Committee members plus Dr K Bannister (Adelaide, Australia), Dr J Beutler (Utrecht, The Netherlands), Professor D Chae (Kyungki-Do, South Korea), Professor SM Cobbe (Glasgow, UK), Dr B Espinoza Vazquez (Colonia Toriello Guerra, Mexico), Professor C Gronhagen-Riska (Helsinki, Finland), Dr J Lima (Sao Paulo, Brazil), Professor R Lins (Antwerpen, Belgium), Dr A McMahon (Edmonton, Canada), Professor G Mayer (Innsbruck, Austria), Professor H Parving (Gentofte, Denmark), Professor G Remuzzi (Bergamo, Italy), Dr O Samuelsson (Goteborg, Sweden), Professor S Sonkodi (Szeged, Hungary), Dr G Suleymanlar (Antalya, Turkey), Professor V Tesar (Prague, Czech Republic), Dr D Tsakiris (Veria, Greece), Professor V Todorov (Pleven, Bulgaria), Professor A Wiecek (Katowice, Poland), Professor R Wûthrich (Gallen, Switzerland).

### Data and Safety Monitoring Board

Professor H Dargie (Chair; Glasgow, UK), Professor E Ritz (Heidelberg, Germany), Professor H Wedel (Goteborg, Sweden), Professor AH Zwinderman (Amsterdam, The Netherlands).

### Clinical Endpoint Committee

Professor SM Cobbe (Chair; Glasgow, UK), Dr A Brady (Glasgow, UK), Dr C Deighan (Glasgow, UK), Dr A Gaw (Glasgow, UK), Professor P Macfarlane (Glasgow, UK), Professor D Stott (Glasgow, UK).

## Supplementary Material

Additional File 1 Table 2. study plan (includes table footnotes) Click here for file
