# Supplementary material for: Effect of rosuvastatin on outcomes in chronic haemodialysis patients – design and rationale of the AURORA study
Source: Curr Control Trials Cardiovasc Med. 2005 May 23;6(1):9. doi: 10.1186/1468-6708-6-9 (PMC1175096; doi:10.1186/1468-6708-6-9)
Supplement: Additional File 1 — Table 2. study plan (includes table footnotes) [file 1468-6708-6-9-S1.pdf]

Table 2: Study plan

	Screening	Randomised treatment phase										
**Visit**	**1**	**2**	**3**	**4**	**5**	**6**	**7**	**8**	**9**	**10**	**Every 6 months[a]**	**Final visit[b]**
**Month**	**–14 days**	**0**	**3**	**6**	**12**	**18**	**24**	**30**	**36**	**42**		
Informed consent	✓											
Medical history	✓											
Inclusion/exclusion criteria	✓											
Physical examination[c]	✓				✓[d]							✓
Clinical chemistry[e]	✓				✓[f]							✓[g]
CK and ALT[h,i]	✓		j	j	✓	j	✓	j	✓	j	✓	✓
Lipid profile and hsCRP[k]	✓		✓		✓		✓[l]		✓[l]		✓[l]	✓[l]
Dialysis data[m]	✓				✓							
AE review		✓	✓	✓	✓	✓	✓	✓	✓	✓	✓	✓
Endpoint review			✓	✓	✓	✓	✓	✓	✓	✓	✓	✓
Study drug dispensing		✓		✓	✓	✓	✓	✓	✓	✓	✓	✓
Study drug compliance				✓	✓	✓	✓	✓	✓	✓	✓	✓
Concomitant medications	✓	✓	✓	✓	✓	✓	✓	✓	✓	✓	✓	✓

[a]If the study is still ongoing, further check-ups will be performed every 6 months; [b]The final visit will occur when 620 subjects with major cardiovascular events have been recorded and also if the study is terminated early; [c]Resting heart rate, blood pressure, height and weight to be measured after the dialysis session; [d]Blood pressure only to be measured at this visit; [e]Performed at the central laboratory and including TSH (at baseline only) and complete blood count measured pre-dialysis; [f]Haemoglobin, haematocrit, albumin, calcium and phosphate only; [g]Complete clinical chemistry and blood count only; [h]Pre-dialysis CK and ALT to be measured at 12 monthly intervals at central laboratory; [i]CK may be measured locally if subject suffers from muscle pains at any time during the study; [j]Pre-dialysis ALT measured locally every 12 months when not assessed centrally; [k]TC, LDL-C, HDL-C, non-HDL-C, TC/HDL-C, LDL-C/HDL-C, TG, Apo B, Apo AI, Apo B/Apo AI ratio, oxidised LDL and hsCRP will be measured by a central laboratory. Subjects should fast for 6 hours prior to lipid assessment pre-dialysis; [l]Only changes in TC, LDL-C, HDL-C and TG to be measured; [m]Pre- and post-urea measurements to calculate dialysis adequacy, method and duration (hours/week)

ALT: Alanine transaminase, **AE:** Adverse events, **Apo AI:** Apolipoprotein AI, **Apo B:** Apolipoprotein B, **CK:** Creatine kinase, **HDL-C:** High-density lipoprotein cholesterol, **hsCRP:** High-sensitivity C-reactive protein, **LDL-C:** Low-density lipoprotein cholesterol, **TC:** Total cholesterol, **TG:** Triglycerides, **TSH:** Thyroid stimulating hormone
